# Aqua­(6,6′-oxydipicolinato-κ^2^ *O*,*N*,*N*′,*O*′)copper(II)

**DOI:** 10.1107/S160053680905346X

**Published:** 2009-12-16

**Authors:** Jingya Sun, Xiangdi Tong

**Affiliations:** a College of Marine Sciences, Zhejiang Ocean University, Zhoushan 316000, People’s Republic of China

## Abstract

In the title complex, [Cu(C_12_H_6_N_2_O_5_)(H_2_O)], the Cu^II^ ion is in a slightly distorted square-pyramidal coordination environment with two N and two O atoms from a 6,6′-oxydipicolinate ligand occupying the basal plane and a water ligand in the apical site. The dihedral angle between the two pyridine rings is 5.51 (6)°. In the crystal structure, inter­molecular O—H⋯O hydrogen bonds link mol­ecules into a two-dimensional network. In addition, weak inter­molecular C—H⋯O and C=O(lone pair)⋯π(ring) inter­actions, with O⋯ring-centroid distances of 3.697 (4) and 3.094 (4) Å, provide additional stabilization.

## Related literature

For inter­molecular inter­actions, see: Choudhury *et al.* (2008 ▶). For the applications of picolinic acid compounds, see: Mann *et al.* (1992 ▶).
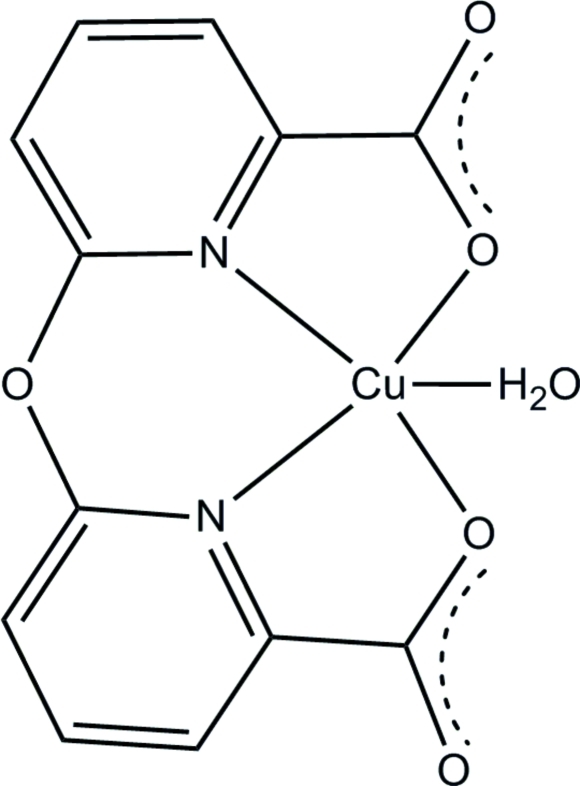


## Experimental

### 

#### Crystal data

[Cu(C_12_H_6_N_2_O_5_)(H_2_O)]
*M* *_r_* = 339.74
Monoclinic, 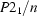

*a* = 7.2487 (16) Å
*b* = 21.055 (4) Å
*c* = 8.2269 (17) Å
β = 110.201 (9)°
*V* = 1178.4 (4) Å^3^
*Z* = 4
Mo *K*α radiation
μ = 1.89 mm^−1^
*T* = 296 K
0.40 × 0.35 × 0.30 mm

#### Data collection

Siemens SMART CCD diffractometer
Absorption correction: multi-scan (*SADABS*; Sheldrick, 1996 ▶) *T*_min_ = 0.519, *T*_max_ = 0.602
6790 measured reflections
2074 independent reflections
1806 reflections with *I* > 2σ(*I*)
*R*_int_ = 0.027

#### Refinement

*R*[*F* ^2^ > 2σ(*F* ^2^)] = 0.032
*wR*(*F* ^2^) = 0.110
*S* = 1.18
2074 reflections
190 parameters
H-atom parameters constrained
Δρ_max_ = 0.48 e Å^−3^
Δρ_min_ = −0.37 e Å^−3^

### 

Data collection: *SMART* (Siemens, 1996 ▶); cell refinement: *SAINT* (Siemens, 1996 ▶); data reduction: *SAINT*; program(s) used to solve structure: *SHELXS97* (Sheldrick, 2008 ▶); program(s) used to refine structure: *SHELXL97* (Sheldrick, 2008 ▶); molecular graphics: *PLATON* (Spek, 2009 ▶) and *SHELXTL* (Sheldrick, 2008 ▶); software used to prepare material for publication: *SHELXTL*.

## Supplementary Material

Crystal structure: contains datablocks I, global. DOI: 10.1107/S160053680905346X/lh2964sup1.cif

Structure factors: contains datablocks I. DOI: 10.1107/S160053680905346X/lh2964Isup2.hkl

Additional supplementary materials:  crystallographic information; 3D view; checkCIF report

## Figures and Tables

**Table 1:** Hydrogen-bond geometry (Å, °)

*D*—H⋯*A*	*D*—H	H⋯*A*	*D*⋯*A*	*D*—H⋯*A*
C8—H8⋯O4^i^	0.93	2.31	3.229 (4)	171
C9—H9⋯O2^ii^	0.93	2.42	3.331 (4)	165
C4—H4⋯O6^iii^	0.93	2.54	3.303 (4)	140
O6—H6*B*⋯O4^iv^	0.85	1.97	2.772 (3)	157
O6—H6*A*⋯O2^v^	0.85	2.01	2.807 (3)	156

Symmetry codes: (i) 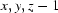; (ii) 
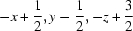
; (iii) 
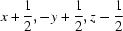
; (iv) 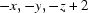; (v) 
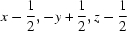
.

**Fig. 1.**
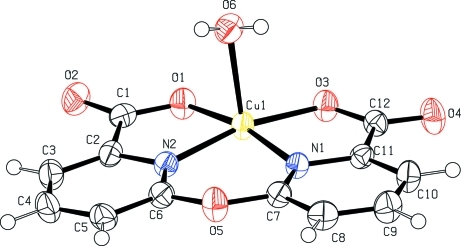
The molecular structure of (I) showing 50% proability displacement ellipsoids and the atom-numbering scheme.

**Fig. 2.**
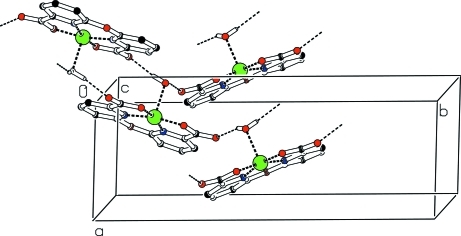
Part of the crystal structure of (I) showing hydrogen bonds as dashed lines.

**Fig. 3.**
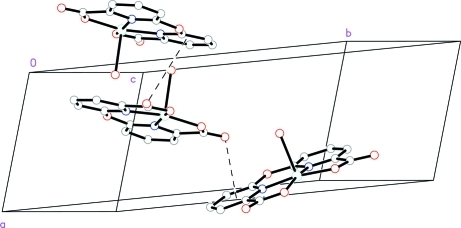
Part of the crystal structure of (I) showing C=O(lone pair)···π(ring) stacking interactions as dashed lines.

## supplementary crystallographic information

### Comment

Picolinic acid compounds play an vital role in the development of coordination chemistry related to catalysis, magnetism and molecular architectures (Mann *et al.*, 1992). As part of our studies on the synthesis and characterization of these types of compounds, we report here the synthesis and crystal structure of the title compound (I).

The molecular structure of the title compound (I) is shown in Fig. 1. The Cu^II^ ion is in a slightly distorted square-pyramidal coordination environment with two N and two O atoms from a 6,6'-oxydipicolinato ligand occupying the basal plane and one water ligand in the apical site. The dihedral angle between the two pyridine rings is 5.51 (6)°. The delocalization of electrons within the carboxylate groups is reflected in the C═O lengths. In the crystal structure, there are intermolecular O—H···O hydrogen bonds involving the carboxyl oxygen atoms and coordinated water molecules (Fig. 2) forming a two-dimensional network (see Table 1 for hydrogen bond geometries). In addition to weak intermolecular C-H···O interactions, further stabilization appears to be provided by weak C=O(lone pair)···π(ring) stacking interactions (Choudhury *et al.*, 2008). The relevant distances are  C12—O4···*Cg1*^i^ = 3.697 (4) Å, *Cg1* is the centroid of the ring defined by the atoms N1/C7-C11 [symmetry code: (i) -x, -y, 2-z] and the angle C12—O4···*Cg1*^i^ is 98.95 (34)°;  C1—O2···*Cg2*^ii^ = 3.094 (4) Å, *Cg2* is the centroid of the ring defined by the atoms N2/C2-C6 [symmetry code: (ii) 0.5+x, 0.5-y, 0.5+z] and the angle C1—O2···*Cg2*^ii^ is 115.48 (4)° (see Fig. 3).

### Experimental

All reagents were available commercially and were used without further purification. 6,6'-Oxydipicolinic acid (260 mg) was added to 1 mmol (132 mg) of CuCl_2_ in 10 ml of water. The suspension was stirred for 4 h and filtered. After leaving the filtrate in air for one week, blue block-shaped crystals of (I) were formed. The crystals were isolated, washed with water three times and dried in a vacuum desicator using silica gel (Yield 75%). Elemental analysis: found C, 42.05; H, 2.96; N, 8.18%; calc. for C_12_H_8_CuN_2_O_6_; C, 42.17; H, 2.95; N, 8.20%.

### Refinement

H atoms bonded to C atoms were positioned geometrically and refined using a riding-model approximation with C–H = 0.93 Å, and *U*_iso_(H) = 1.2*U*_eq_(C). H atoms bonded to O atoms were found in difference Fourier maps and included as riding with O—H = 0.85Å and U_iso_(H) = 1.2*U*_eq_(O).

### Crystal data

**Table:**

[Cu(C_12_H_6_N_2_O_5_)(H_2_O)]	*F*(000) = 684
*M**_r_* = 339.74	*D*_x_ = 1.915 Mg m^−^^3^
Monoclinic, *P*2_1_/*n*	Mo *K*α radiation, λ = 0.71073 Å
Hall symbol: -P 2yn	Cell parameters from 2501 reflections
*a* = 7.2487 (16) Å	θ = 2.8–27.5°
*b* = 21.055 (4) Å	µ = 1.89 mm^−^^1^
*c* = 8.2269 (17) Å	*T* = 296 K
β = 110.201 (9)°	Block, blue
*V* = 1178.4 (4) Å^3^	0.40 × 0.35 × 0.30 mm
*Z* = 4	

### Data collection

**Table:**

Siemens SMART CCD diffractometer	2074 independent reflections
Radiation source: fine-focus sealed tube	1806 reflections with *I* > 2σ(*I*)
graphite	*R*_int_ = 0.027
φ and ω scans	θ_max_ = 25.0°, θ_min_ = 1.9°
Absorption correction: multi-scan (*SADABS*; Sheldrick, 1996)	*h* = −8→8
*T*_min_ = 0.519, *T*_max_ = 0.602	*k* = −24→24
6790 measured reflections	*l* = −9→8

### Refinement

**Table:**

Refinement on *F*^2^	Primary atom site location: structure-invariant direct methods
Least-squares matrix: full	Secondary atom site location: difference Fourier map
*R*[*F*^2^ > 2σ(*F*^2^)] = 0.032	Hydrogen site location: inferred from neighbouring sites
*wR*(*F*^2^) = 0.110	H-atom parameters constrained
*S* = 1.18	*w* = 1/[σ^2^(*F*_o_^2^) + (0.0671*P*)^2^ + 0.1209*P*] where *P* = (*F*_o_^2^ + 2*F*_c_^2^)/3
2074 reflections	(Δ/σ)_max_ < 0.001
190 parameters	Δρ_max_ = 0.48 e Å^−^^3^
0 restraints	Δρ_min_ = −0.37 e Å^−^^3^

### Special details

**Table:**

Geometry. All esds (except the esd in the dihedral angle between two l.s. planes) are estimated using the full covariance matrix. The cell esds are taken into account individually in the estimation of esds in distances, angles and torsion angles; correlations between esds in cell parameters are only used when they are defined by crystal symmetry. An approximate (isotropic) treatment of cell esds is used for estimating esds involving l.s. planes.
Refinement. Refinement of F^2^ against ALL reflections. The weighted R-factor wR and goodness of fit S are based on F^2^, conventional R-factors R are based on F, with F set to zero for negative F^2^. The threshold expression of F^2^ > 2sigma(F^2^) is used only for calculating R-factors(gt) etc. and is not relevant to the choice of reflections for refinement. R-factors based on F^2^ are statistically about twice as large as those based on F, and R- factors based on ALL data will be even larger.

### Fractional atomic coordinates and isotropic or equivalent isotropic displacement parameters (Å^2^)

**Table:**

	*x*	*y*	*z*	*U*_iso_*/*U*_eq_	
Cu1	0.33165 (5)	0.109980 (17)	0.96148 (4)	0.02725 (18)	
O3	0.2907 (3)	0.05841 (10)	1.1417 (3)	0.0327 (5)	
N2	0.4163 (4)	0.14875 (12)	0.7848 (3)	0.0265 (6)	
O1	0.4318 (3)	0.18774 (11)	1.0899 (3)	0.0336 (5)	
O5	0.3272 (4)	0.06644 (11)	0.5777 (3)	0.0368 (6)	
N1	0.2837 (4)	0.03042 (12)	0.8315 (3)	0.0254 (6)	
O4	0.2158 (4)	−0.04040 (11)	1.1935 (3)	0.0409 (6)	
O2	0.5275 (4)	0.28511 (11)	1.0481 (3)	0.0410 (6)	
C12	0.2463 (4)	0.00062 (15)	1.1003 (4)	0.0268 (7)	
C1	0.4833 (5)	0.23022 (15)	1.0031 (4)	0.0297 (7)	
O6	0.0171 (3)	0.14739 (11)	0.8390 (3)	0.0382 (6)	
H6A	−0.0120	0.1623	0.7373	0.046*	
H6B	−0.0521	0.1144	0.8010	0.046*	
C2	0.4850 (5)	0.20891 (14)	0.8261 (4)	0.0284 (7)	
C5	0.4587 (5)	0.15934 (17)	0.5117 (4)	0.0342 (8)	
H5	0.4473	0.1419	0.4048	0.041*	
C11	0.2346 (4)	−0.01770 (15)	0.9187 (4)	0.0258 (7)	
C10	0.1814 (5)	−0.07640 (16)	0.8462 (4)	0.0348 (8)	
H10	0.1481	−0.1089	0.9075	0.042*	
C6	0.4036 (5)	0.12555 (15)	0.6311 (4)	0.0298 (7)	
C8	0.2272 (5)	−0.03744 (16)	0.5894 (4)	0.0358 (8)	
H8	0.2241	−0.0430	0.4763	0.043*	
C7	0.2808 (5)	0.02011 (15)	0.6721 (4)	0.0280 (7)	
C9	0.1787 (5)	−0.08608 (16)	0.6763 (4)	0.0374 (8)	
H9	0.1441	−0.1255	0.6235	0.045*	
C3	0.5440 (5)	0.24521 (16)	0.7162 (4)	0.0366 (8)	
H3	0.5919	0.2861	0.7470	0.044*	
C4	0.5311 (5)	0.21967 (17)	0.5561 (5)	0.0407 (9)	
H4	0.5715	0.2435	0.4793	0.049*	

### Atomic displacement parameters (Å^2^)

**Table:**

	*U*^11^	*U*^22^	*U*^33^	*U*^12^	*U*^13^	*U*^23^
Cu1	0.0409 (3)	0.0226 (3)	0.0216 (3)	−0.00293 (15)	0.01511 (19)	−0.00212 (14)
O3	0.0481 (14)	0.0300 (13)	0.0229 (11)	−0.0044 (10)	0.0161 (10)	−0.0017 (9)
N2	0.0309 (14)	0.0253 (14)	0.0242 (13)	0.0005 (11)	0.0105 (10)	0.0002 (11)
O1	0.0470 (14)	0.0288 (12)	0.0267 (11)	−0.0051 (10)	0.0150 (10)	−0.0053 (9)
O5	0.0589 (16)	0.0326 (13)	0.0237 (11)	−0.0090 (11)	0.0203 (11)	−0.0052 (10)
N1	0.0327 (14)	0.0225 (13)	0.0220 (12)	−0.0009 (11)	0.0108 (10)	−0.0010 (10)
O4	0.0594 (16)	0.0381 (14)	0.0303 (12)	−0.0093 (12)	0.0219 (11)	0.0034 (11)
O2	0.0495 (15)	0.0263 (13)	0.0471 (15)	−0.0071 (10)	0.0166 (12)	−0.0104 (11)
C12	0.0283 (16)	0.0302 (18)	0.0215 (15)	0.0000 (13)	0.0082 (13)	0.0033 (13)
C1	0.0275 (16)	0.0285 (19)	0.0323 (16)	0.0031 (13)	0.0094 (13)	−0.0018 (14)
O6	0.0378 (13)	0.0339 (13)	0.0400 (13)	0.0015 (10)	0.0098 (11)	0.0019 (11)
C2	0.0286 (16)	0.0233 (16)	0.0310 (16)	−0.0001 (13)	0.0075 (13)	0.0014 (13)
C5	0.0361 (18)	0.040 (2)	0.0298 (17)	0.0017 (14)	0.0164 (14)	0.0037 (14)
C11	0.0289 (16)	0.0250 (16)	0.0234 (15)	0.0026 (12)	0.0087 (12)	0.0014 (12)
C10	0.048 (2)	0.0268 (18)	0.0324 (18)	−0.0054 (14)	0.0167 (15)	−0.0001 (14)
C6	0.0361 (18)	0.0305 (18)	0.0246 (16)	0.0025 (14)	0.0130 (13)	0.0022 (13)
C8	0.046 (2)	0.036 (2)	0.0277 (16)	−0.0008 (15)	0.0161 (15)	−0.0095 (15)
C7	0.0348 (17)	0.0279 (17)	0.0227 (15)	0.0012 (13)	0.0117 (13)	−0.0001 (13)
C9	0.050 (2)	0.0285 (18)	0.0346 (18)	−0.0074 (16)	0.0160 (16)	−0.0114 (15)
C3	0.0374 (19)	0.0310 (18)	0.042 (2)	−0.0050 (15)	0.0142 (15)	0.0034 (16)
C4	0.043 (2)	0.042 (2)	0.042 (2)	−0.0028 (16)	0.0217 (16)	0.0123 (17)

### Geometric parameters (Å, °)

**Table:**

Cu1—O3	1.942 (2)	O6—H6A	0.8500
Cu1—N2	1.942 (3)	O6—H6B	0.8501
Cu1—O1	1.948 (2)	C2—C3	1.361 (5)
Cu1—N1	1.953 (2)	C5—C4	1.375 (5)
Cu1—O6	2.290 (2)	C5—C6	1.379 (4)
O3—C12	1.275 (4)	C5—H5	0.9300
N2—C6	1.328 (4)	C11—C10	1.368 (5)
N2—C2	1.361 (4)	C10—C9	1.406 (5)
O1—C1	1.278 (4)	C10—H10	0.9300
O5—C7	1.359 (4)	C8—C9	1.363 (5)
O5—C6	1.371 (4)	C8—C7	1.378 (5)
N1—C7	1.323 (4)	C8—H8	0.9300
N1—C11	1.358 (4)	C9—H9	0.9300
O4—C12	1.225 (4)	C3—C4	1.395 (5)
O2—C1	1.222 (4)	C3—H3	0.9300
C12—C11	1.517 (4)	C4—H4	0.9300
C1—C2	1.528 (4)		
			
O3—Cu1—N2	167.91 (10)	N2—C2—C1	112.9 (3)
O3—Cu1—O1	100.51 (9)	C3—C2—C1	125.2 (3)
N2—Cu1—O1	84.13 (10)	C4—C5—C6	117.7 (3)
O3—Cu1—N1	83.87 (9)	C4—C5—H5	121.1
N2—Cu1—N1	89.62 (10)	C6—C5—H5	121.1
O1—Cu1—N1	168.68 (10)	N1—C11—C10	122.0 (3)
O3—Cu1—O6	97.86 (10)	N1—C11—C12	113.2 (3)
N2—Cu1—O6	92.86 (10)	C10—C11—C12	124.8 (3)
O1—Cu1—O6	94.45 (9)	C11—C10—C9	118.0 (3)
N1—Cu1—O6	95.29 (10)	C11—C10—H10	121.0
C12—O3—Cu1	114.87 (19)	C9—C10—H10	121.0
C6—N2—C2	118.7 (3)	N2—C6—O5	121.8 (3)
C6—N2—Cu1	128.4 (2)	N2—C6—C5	123.1 (3)
C2—N2—Cu1	112.8 (2)	O5—C6—C5	115.1 (3)
C1—O1—Cu1	114.29 (19)	C9—C8—C7	118.8 (3)
C7—O5—C6	128.3 (2)	C9—C8—H8	120.6
C7—N1—C11	118.9 (3)	C7—C8—H8	120.6
C7—N1—Cu1	128.5 (2)	N1—C7—O5	121.8 (3)
C11—N1—Cu1	112.4 (2)	N1—C7—C8	122.6 (3)
O4—C12—O3	126.1 (3)	O5—C7—C8	115.6 (3)
O4—C12—C11	118.5 (3)	C8—C9—C10	119.7 (3)
O3—C12—C11	115.4 (3)	C8—C9—H9	120.2
O2—C1—O1	126.1 (3)	C10—C9—H9	120.2
O2—C1—C2	118.6 (3)	C2—C3—C4	118.5 (3)
O1—C1—C2	115.3 (3)	C2—C3—H3	120.8
Cu1—O6—H6A	115.6	C4—C3—H3	120.8
Cu1—O6—H6B	104.6	C5—C4—C3	120.1 (3)
H6A—O6—H6B	91.5	C5—C4—H4	119.9
N2—C2—C3	121.9 (3)	C3—C4—H4	119.9

### Hydrogen-bond geometry (Å, °)

**Table:**

*D*—H···*A*	*D*—H	H···*A*	*D*···*A*	*D*—H···*A*
C8—H8···O4^i^	0.93	2.31	3.229 (4)	171
C9—H9···O2^ii^	0.93	2.42	3.331 (4)	165
C4—H4···O6^iii^	0.93	2.54	3.303 (4)	140
O6—H6B···O4^iv^	0.85	1.97	2.772 (3)	157
O6—H6A···O2^v^	0.85	2.01	2.807 (3)	156

Symmetry codes: (i) *x*, *y*, *z*−1; (ii) −*x*+1/2, *y*−1/2, −*z*+3/2; (iii) *x*+1/2, −*y*+1/2, *z*−1/2; (iv) −*x*, −*y*, −*z*+2; (v) *x*−1/2, −*y*+1/2, *z*−1/2.
